# Heart Rate Recovery Index and Pulse Wave Velocity in HFpEF: Exploratory Cutoff Values from a Cross-Sectional Study

**DOI:** 10.3390/jcm15218363

**Published:** 2026-10-27

**Authors:** Bogdan-Simion Suciu, Oana Pătru, Silvia Luca, Adelina Andreea Faur-Grigori, Bogdan Enache, Dragoș Cozma

**Affiliations:** 1 Doctoral School, “Victor Babes” University of Medicine and Pharmacy, Eftimie Murgu Square 2, 300041 Timisoara, Romania; bogdan.suciu@umft.ro (B.-S.S.); silvia.luca@umft.ro (S.L.); andreea.faur@umft.ro (A.A.F.-G.); 2 Institute of Cardiovascular Diseases Timisoara, 13A Gheorghe Adam Street, 300310 Timisoara, Romania; bogdan.enache@umft.ro (B.E.); dragos.cozma@umft.ro (D.C.); 3 Research Center of the Institute of Cardiovascular Diseases Timisoara, 13A Gheorghe Adam Street, 300310 Timisoara, Romania; 4 Cardiology Department, “Victor Babes” University of Medicine and Pharmacy, Eftimie Murgu Square 2, 300041 Timisoara, Romania

**Keywords:** heart failure with preserved ejection fraction, pulse wave velocity, heart rate recovery, arterial stiffness, autonomic dysfunction, diagnostic markers

## Abstract

**Background/Objectives**: Heart failure with preserved ejection fraction (HFpEF) remains challenging to diagnose, and established algorithms such as HFA-PEFF and H2FPEF frequently classify patients within intermediate probability categories requiring additional testing. Arterial stiffness and cardiac autonomic dysfunction are increasingly recognized as relevant components of HFpEF pathophysiology, yet the combined discriminatory value of pulse wave velocity (PWV) and heart rate recovery index (HRRI) has not been well established. This study aimed to evaluate the individual and combined discriminatory performance of PWV and HRRI for HFpEF and to assess their incremental contribution beyond established clinical risk factors. **Methods**: In this prospective, single-center, cross-sectional study, 180 participants were included, comprising 86 patients with HFpEF and 94 participants without heart failure. Group allocation was clinically assigned by the treating cardiologist with access to PWV and HRRI results. PWV was assessed non-invasively using an oscillometric device, and HRRI was derived from standardized cycle ergometer exercise testing. Univariable and multivariable logistic regression, receiver operating characteristic (ROC) curve analysis, and likelihood-ratio testing were performed to assess independent associations and incremental model contribution. Sensitivity analyses evaluated the influence of age modelling and beta-blocker use. **Results**: Both PWV and HRRI were independently associated with clinically assigned HFpEF status after adjustment for age, sex, body mass index, hypertension, diabetes mellitus, dyslipidemia, and atrial fibrillation (adjusted OR 1.330 per 1 m/s increase in PWV and 0.857 per 0.1-unit increase in HRRI; both *p* ≤ 0.023). PWV and HRRI demonstrated AUCs of 0.770 and 0.792, respectively, with Youden index-derived cutoff values of ≥9.60 m/s and ≤1.97. Their combination significantly improved discrimination compared with either marker alone (AUC 0.833). Adding both markers to the clinical model improved model fit, but the AUC increase from 0.865 to 0.896 was not statistically significant after Holm adjustment (adjusted *p* = 0.132). The associations of both markers with HFpEF remained essentially unchanged after adjustment for beta-blocker therapy. **Conclusions**: PWV and HRRI were associated with clinically assigned HFpEF status; the observed discrimination does not establish independently validated diagnostic accuracy. Incremental discrimination beyond clinical characteristics and clinical usefulness remain to be established. The cutoff values are exploratory and should not be used as clinical decision thresholds without formal measurement reliability assessment and independent prospective validation.

## 1. Introduction

Heart failure (HF) is a complex clinical syndrome affecting approximately 1–3% of the global population and is considered one of the leading causes of cardiovascular morbidity and mortality worldwide [1].

The 2026 European Society of Cardiology guidelines classify HF into two phenotypes according to left ventricular ejection fraction (LVEF): HF with reduced ejection fraction (HFrEF; LVEF < 50%) and HF with preserved ejection fraction (HFpEF; LVEF ≥ 50%) [2].

Among its subtypes, HFpEF is characterized by symptoms and signs of HF, an LVEF ≥ 50%, and objective evidence of structural and/or functional cardiac abnormalities consistent with left ventricular diastolic dysfunction or elevated filling pressures. It accounts for approximately half of all HF cases, and its prevalence continues to increase in parallel with rising life expectancy [3,4,5].

The diagnosis of HFpEF remains challenging and requires an integrated clinical assessment [6]. Current guidelines recommend a combination of clinical parameters, echocardiographic findings, and biomarkers such as natriuretic peptides to aid in establishing the diagnosis [7,8,9,10]. However, natriuretic peptide concentrations may be below diagnostic thresholds in some patients with HFpEF, particularly those with obesity [11], and resting echocardiographic findings may be inconclusive [6]. Two diagnostic algorithms have been developed to support the diagnosis of HFpEF: the HFA-PEFF and H_2_FPEF algorithms [6,12]. Echocardiography also provides noninvasive hemodynamic information [13].

The H_2_FPEF score, developed by Reddy et al., integrates six clinical and echocardiographic variables, obesity, atrial fibrillation, age > 60 years, treatment with ≥2 antihypertensive medications, E/e′ ratio > 9, and pulmonary artery systolic pressure > 35 mmHg, into a composite score, achieving an area under the receiver operating characteristic curve of 0.841 in the derivation cohort [14].

The HFA-PEFF algorithm, published by the Heart Failure Association of the European Society of Cardiology in 2019, proposes a stepwise diagnostic approach in which a score of ≥5 points is diagnostic of HFpEF, a score of ≤1 makes the diagnosis very unlikely, and intermediate scores of 2–4 require further workup, including diastolic stress echocardiography or invasive hemodynamic assessment [6].

Despite their clinical utility, both algorithms leave a proportion of patients in intermediate probability categories. Neither incorporates direct measures of arterial stiffness or exercise-derived heart rate recovery. These limitations motivate the investigation of complementary non-invasive markers, such as pulse wave velocity (PWV) and heart rate recovery index (HRRI), that might provide additional information within current diagnostic frameworks.

Contemporary research has increasingly recognized HFpEF as a systemic disorder involving vascular and autonomic abnormalities [15,16]. Arterial stiffness, commonly quantified non-invasively by PWV, is increasingly recognized as an important factor in HFpEF pathophysiology, contributing to increased left ventricular afterload and promoting diastolic dysfunction [17]. Population-based longitudinal data are consistent with this proposed link: in a large cohort study, estimated PWV was significantly associated with an increased risk of new-onset HF [18]. A recent systematic review and meta-analysis also found an association between arterial stiffness assessed by PWV and HF, although its prognostic value specifically in HFpEF remains incompletely defined [19]. Long-term data from the Framingham Heart Study further demonstrated an association between arterial stiffness and adverse cardiovascular outcomes independent of traditional risk factors [20]. Mechanistically, arterial stiffness has been proposed to contribute to HFpEF through several interconnected pathways, including increased pulse pressure, premature wave reflection, and impaired ventricular–arterial coupling, although the precise causal relationship and its clinical implications continue to be investigated [21].

In parallel with vascular abnormalities, autonomic dysfunction has been implicated in HFpEF, with chronotropic incompetence during exercise and delayed post-exercise heart rate recovery among its manifestations [15]. A study comparing patients with HFpEF, healthy controls, and hypertensive controls demonstrated impaired chronotropic responses and abnormal heart rate recovery in patients with HFpEF. Hypertensive controls showed chronotropic responses and heart rate recovery similar to those of healthy controls, suggesting that these abnormalities may not be explained by hypertension alone [22].

HRRI, defined as the ratio of heart rate acceleration time during exercise to deceleration time during post-exercise recovery, integrates the temporal characteristics of the exercise and recovery heart rate responses and may provide complementary information in the assessment of patients with HFpEF [15,23].

This study aimed to assess the associations of PWV and HRRI with HFpEF and to evaluate their individual and combined discriminative performance in participants with and without HF. We also examined their incremental value beyond established clinical risk factors and derived exploratory cutoff values within the study sample.

## 2. Materials and Methods

### 2.1. Study Design and Study Population

This was a prospective, observational, single-center, cross-sectional study including patients with HFpEF and participants without heart failure (non-HF group). Participants were prospectively enrolled at the Institute of Cardiovascular Diseases Timișoara, Romania, between November 2025 and August 2026.

Participants were assessed consecutively during the recruitment period. All individuals who met the eligibility criteria and provided study-specific consent were enrolled. The HFpEF group included adults aged ≥18 years with a diagnosis of HFpEF and a left ventricular ejection fraction (LVEF) ≥ 50% who were clinically stable at the time of study assessment. The non-HF comparator group comprised individuals undergoing cardiovascular evaluation, commonly for blood pressure assessment, chest pain, or palpitations, rather than healthy population-based controls. Cardiovascular risk factors, including hypertension and dyslipidemia, were therefore present in this group.

HFpEF was diagnosed in accordance with European Society of Cardiology recommendations through an integrated assessment of symptoms and/or signs of HF, LVEF ≥ 50%, and objective echocardiographic evidence of structural and/or functional abnormalities consistent with elevated left ventricular filling pressures, supported by NT-proBNP when available. The H_2_FPEF score and available components of the HFA-PEFF algorithm informed the assessment but were not sole determinants of group allocation. NT-proBNP was available in 153/180 participants (85.0%). For the remaining 27 participants, the biomarker domain could not be evaluated; these participants are reported separately in the HFA-PEFF probability summary. Participants with intermediate diagnostic probability or incomplete HFA-PEFF assessment were classified by the treating cardiologist using the available clinical, echocardiographic, and laboratory findings. The non-HF group comprised participants without a clinical heart failure diagnosis after cardiovascular evaluation. Diastolic stress echocardiography and invasive hemodynamic assessment were not performed systematically. The treating cardiologist had access to PWV and HRRI results when final group allocation was recorded.

Following written study-specific informed consent, participants underwent a standardized evaluation comprising clinical assessment, resting 12-lead electrocardiography, routine-care laboratory testing and echocardiography, and protocol-specified cycle ergometer testing for HRRI assessment and PWV measurement. Echocardiography and routine laboratory testing were performed as part of clinical care, whereas PWV measurement and cycle ergometer testing for HRRI assessment were specified by the research protocol.

The study protocol was approved by the local Ethics Committee of the Institute of Cardiovascular Diseases Timișoara (approval No. 82/03.11.2025) and was conducted in accordance with the principles of the Declaration of Helsinki. Written informed consent for study participation was obtained using a dedicated form, separate from the general admission consent, before study procedures.

### 2.2. Inclusion Criteria

Eligible participants were adults aged ≥18 years who provided written informed consent and had a complete clinical assessment, including medical history, cardiovascular risk factors, current pharmacological treatment, and transthoracic echocardiography of adequate image quality for the assessment of LVEF and relevant structural and functional parameters.

For inclusion in the HFpEF group, participants were required to have a diagnosis of heart failure with preserved ejection fraction established according to contemporary guideline recommendations, an LVEF ≥ 50%, and clinical stability at the time of study evaluation.

For inclusion in the non-HF group, participants were required to have no clinical diagnosis of heart failure and to undergo the same study-specific cardiovascular evaluation. These participants were recruited among individuals admitted for day hospitalization at the Institute of Cardiovascular Diseases Timișoara who agreed to participate in this study.

### 2.3. Exclusion Criteria

Exclusion criteria were age < 18 years; left ventricular ejection fraction < 50%; life-threatening ventricular arrhythmias; high-grade or complete atrioventricular block; significant rhythm disturbances that could interfere with the assessment of heart rate dynamics during exercise, including atrial fibrillation at the time of exercise testing; severe aortic stenosis; congenital heart disease; active malignancy with major short-term prognostic impact; recent acute clinical events, including acute coronary syndrome or severe infection; peripheral arterial disease or other conditions likely to significantly interfere with pulse wave velocity assessment; and inability to perform bicycle ergometer exercise testing because of contraindications, severe orthopedic limitations, or other conditions precluding adequate exercise testing.

### 2.4. Clinical and Demographic Data

Clinical and demographic data were collected at the time of study evaluation and included age, sex, body weight, height, body mass index (BMI), blood pressure, and resting heart rate. Blood pressure and heart rate were measured after at least 5 min of rest, in accordance with current recommendations. A standard 12-lead electrocardiogram was obtained in all participants.

In participants with implantable cardiac devices, device interrogation was performed before exercise testing to document relevant programming parameters, including pacing mode, rate-response settings, and atrioventricular delay.

Medical history and cardiovascular comorbidities were recorded from patient interviews and available medical records. The following conditions were specifically documented: arterial hypertension, coronary artery disease, diabetes mellitus, chronic kidney disease, dyslipidemia, obesity, and previous hospitalization for heart failure. Signs and symptoms present at the time of study evaluation were also recorded.

Current pharmacological treatment was documented for all participants, with particular attention to medications potentially affecting heart rate response during exercise, including beta-blockers, non-dihydropyridine calcium channel blockers, and ivabradine. Use of renin–angiotensin–aldosterone system inhibitors, diuretics, antiplatelet agents, and lipid-lowering therapies was also recorded.

In the HFpEF group, functional status was assessed according to the New York Heart Association (NYHA) functional classification. Laboratory data were obtained from routine blood tests performed during day-case or inpatient evaluation. NT-proBNP values were recorded when available.

### 2.5. Exercise Test

Exercise testing was performed using a semi-recumbent cycle ergometer (Ergoselect 1200, Ergoline GmbH, Bitz, Germany). Before testing, resting blood pressure and heart rate were measured, and participants underwent 12-lead electrocardiography and transthoracic echocardiography. Relevant laboratory data obtained during the clinical evaluation were also recorded.

The incremental workload protocol began at 25 W and increased by 25 W every 2 min. Blood pressure was measured at each stage using the system integrated into the ergometer. Heart rhythm and heart rate were monitored continuously by 12-lead electrocardiography. In participants with implanted rate-adaptive pacemakers, the devices were interrogated before testing. Individualized clinical settings were maintained during the test; no study-specific reprogramming was performed.

Exercise continued until symptom-limited exhaustion or a clinical indication for termination, including limiting dyspnea, chest pain, leg discomfort, clinically significant arrhythmia, or an excessive blood pressure response. Recovery consisted of 1 min of active recovery followed by passive observation until the operator judged that heart rate had approached its pre-exercise value. No numerical tolerance around baseline heart rate or fixed maximum observation period was prespecified. A deceleration-time endpoint was recorded for every participant.

The recorded exercise variables included acceleration time (AT), deceleration time (DT), heart rate recovery index (HRRI), heart rate recovery at 1 min (HRR1), blood pressure at each exercise stage, maximal exercise capacity expressed in metabolic equivalents (METs), peak heart rate, and age-predicted maximal heart rate. AT was defined as the interval from exercise initiation to peak heart rate. DT was defined as the interval from peak heart rate to the operator-defined near-baseline endpoint. Both times were identified manually from the recorded heart-rate curves, and HRRI was calculated as AT/DT. HRR1 was calculated as peak heart rate minus heart rate measured 1 min after exercise cessation.

No repeated exercise tests were performed; therefore, test–retest repeatability of HRRI was not assessed.

### 2.6. Measurement of Pulse Wave Velocity

Pulse wave velocity (PWV) was assessed non-invasively using an oscillometric Arteriograph device (TensioMed Ltd., Budapest, Hungary), in accordance with the manufacturer’s recommendations. Measurements were performed in a quiet, temperature-controlled room at an ambient temperature of 20–23 °C.

Before the examination, participants were instructed to abstain from alcohol consumption, smoking, food intake, and energy drinks for at least 3 h. Participants rested in the supine position for 5–10 min before measurement. Arm circumference and the distance between the suprasternal notch and the pubic symphysis were measured according to the device protocol. The appropriate cuff size was selected based on arm circumference and placed on the upper arm.

During PWV assessment, participants remained supine and were instructed to remain still and refrain from speaking. At least two technically adequate measurements were obtained for each participant during the same examination session. The consistency of repeated measurements was assessed by the operator based on technical adequacy and agreement between the recorded values, without applying a predefined numerical tolerance. When measurements were considered consistent, the mean value was used for statistical analysis. PWV was expressed in meters per second (m/s).

### 2.7. Echocardiographic Assessment

Transthoracic echocardiography was performed in all participants using a VIVID 9 ultrasound system (GE Healthcare, Milwaukee, WI, USA). Standard parasternal long- and short-axis views and apical two-, three-, and four-chamber views were acquired.

Left ventricular ejection fraction (LVEF) was assessed using the biplane Simpson method, and an LVEF ≥ 50% was required for study inclusion. Left ventricular dimensions, interventricular septal thickness, and posterior wall thickness were measured. Longitudinal systolic function was assessed using mitral annular plane systolic excursion (MAPSE) and systolic mitral annular velocity (s′) measured at the septal annulus by tissue Doppler imaging. Left atrial volume was measured and indexed to body surface area to obtain the left atrial volume index (LAVI).

Diastolic function was assessed using pulsed-wave Doppler and tissue Doppler imaging. Early (E) and late (A) diastolic trans-mitral flow velocities were recorded, and the E/A ratio was calculated. Early diastolic mitral annular velocities (e′) were measured at the septal and lateral annulus, and their average was used to calculate the E/e′ ratio. When feasible, peak tricuspid regurgitation velocity was measured to estimate pulmonary artery systolic pressure.

### 2.8. Statistical Analysis

Statistical analysis was performed using MedCalc Statistical Software version 12.5 (MedCalc Software Ltd., Ostend, Belgium). Continuous variables were expressed as median (interquartile range—IQR) and categorical variables as number and percentage. Continuous variables were compared between participants with HFpEF and those without HF using the Mann–Whitney U test. Categorical variables were compared using Pearson’s chi-square test when all expected cell counts were >5; otherwise, Fisher’s exact test was used. All statistical tests were two-sided, and a *p* value < 0.05 was considered statistically significant.

HFpEF was used as the binary dependent variable in logistic regression analyses (HFpEF = 1). Univariable logistic regression was initially performed for age, male, sex, BMI, hypertension, type 2 diabetes mellitus, dyslipidemia, atrial fibrillation, PWV, and HRRI. A multivariable logistic regression model was subsequently conducted including age, male, sex, BMI, hypertension, type 2 diabetes mellitus, dyslipidemia, atrial fibrillation, PWV, and HRRI. Odds ratios (ORs) with 95% confidence intervals (CIs) were reported. PWV was entered as a continuous variable per 1 m/s increase, whereas HRRI was expressed per 0.1-unit increase to facilitate clinical interpretation. Chronic kidney disease was not included in the primary multivariable model because of complete separation resulting from the absence of participants with chronic kidney disease in the non-HF group.

The association between PWV and HRRI was assessed using Spearman’s rank correlation coefficient. Multicollinearity among predictors included in the multivariable logistic regression model was assessed using variance inflation factors (VIFs). Linearity of continuous predictors in the logit was assessed using the Box–Tidwell approach. Model calibration was assessed using the Hosmer–Lemeshow goodness-of-fit test.

As a prespecified sensitivity analysis, the multivariable model was additionally adjusted for beta-blocker use to assess potential confounding, given its established influence on heart rate dynamics.

The discriminatory performance of PWV, HRRI, and their combination for HFpEF was evaluated using receiver operating characteristic (ROC) curve analysis. The area under the ROC curve (AUC) with 95% CI was calculated. Exploratory thresholds for PWV and HRRI were determined using the Youden index, and sensitivity and specificity were calculated at the corresponding thresholds. For the combined PWV + HRRI model, the exploratory threshold was derived from the model-predicted probability using the Youden index. The AUCs of PWV and HRRI were recalculated after excluding participants with chronic kidney disease and after restricting the cohort to participants with measured NT-proBNP. The 95% CIs for the chronic kidney disease-restricted AUCs were calculated using the DeLong method. For the NT-proBNP-available subgroup, 95% percentile CIs were obtained from 5000 nonparametric bootstrap resamples stratified by clinical group.

Two-sided 95% CIs for sensitivity and specificity were calculated using the Wilson score method without continuity correction. The Youden thresholds were selected and evaluated in the same cohort. These threshold-based estimates represent apparent performance; their confidence intervals do not account for threshold-selection or model-development uncertainty.

To estimate optimism in the AUC of the final clinical + PWV + HRRI logistic regression model, 1000 ordinary participant-level bootstrap samples of size 180 were drawn with replacement. The model included age, male sex, body mass index, hypertension, type 2 diabetes mellitus, dyslipidemia, atrial fibrillation, PWV, and HRRI. In each resample, the intercept and all regression coefficients were re-estimated using this fixed predictor set; no predictor selection was performed. The refitted model was evaluated in both the bootstrap sample and the original cohort. Mean optimism was the average difference between these two AUCs and was subtracted from the original apparent AUC. Youden threshold selection was not repeated in the bootstrap samples, and threshold-specific sensitivity and specificity were not internally validated.

## 3. Results

### 3.1. Study Population and Baseline Characteristics

The final cohort comprised 180 participants (86 with HFpEF and 94 without HF). PWV and HRRI were available for all 180. Patients with HFpEF were significantly older than participants without HF, with a median age of 64.00 [56.25–70.75] years versus 48.50 [40.25–57.75] years, respectively (*p* < 0.001). The groups did not differ significantly with respect to sex distribution (*p* = 0.064) or BMI (*p* = 0.124) (Table 1).

Cardiovascular risk factors and comorbidities were more frequent among patients with HFpEF. Hypertension, type 2 diabetes mellitus, and dyslipidemia were significantly more prevalent in the HFpEF group than in participants without HF (all *p* < 0.001). Atrial fibrillation was also more frequent among patients with HFpEF (23/86, 26.74% vs. 6/94, 6.38%; *p* < 0.001). Chronic kidney disease was present in 10/86 (11.63%) patients with HFpEF and in none of the participants without HF (*p* < 0.001). Obesity was more frequent in the HFpEF group (35/85, 41.18% vs. 25/94, 26.60%; *p* = 0.039), although obesity status was missing for one patient with HFpEF. Beta-blocker use was recorded in 101/180 participants (56.11%). Use was more frequent in the HFpEF group than among participants without HF (Table 1).

Beta-blocker users had numerically lower HRRI, higher PWV, and were numerically older than non-users, although none of these differences reached statistical significance (HRRI:1.90 [1.57–2.25] vs. 2.05 [1.67–2.42], *p* = 0.099; PWV: 9.90 [8.50–11.10] vs. 9.20 [7.95–10.80] m/s, *p* = 0.086; age: 57.0 [48.0–67.0] vs. 54.0 [40.5–65.0] years, *p* = 0.063).

NT-proBNP was available in 153 of 180 participants (85.0%): 82 of 86 participants with HFpEF and 71 of 94 non-HF comparators. Objective echocardiographic and biomarker findings are summarized in Table 2. Among participants with measured NT-proBNP, the recorded HFA-PEFF category was high in 47/82 (57.3%) and intermediate in 35/82 (42.7%) of the HFpEF group; it was low in 39/71 (54.9%) and intermediate in 32/71 (45.1%) of the non-HF group. The recorded H_2_FPEF category was intermediate in 62/86 (72.1%) and high in 24/86 (27.9%) participants with HFpEF. Among non-HF comparators, it was low in 34/94 (36.2%) and intermediate in 60/94 (63.8%).

### 3.2. PWV and HRRI

PWV and HRRI differed significantly between participants with HFpEF and those without HF (Table 3). PWV was higher in patients with HFpEF, whereas HRRI was lower compared with those without HF (both *p* < 0.001). These differences are illustrated in Figure 1 and Figure 2.

Exercise-test characteristics are presented in Table S1. Compared with non-HF participants, those with HFpEF had a shorter AT, while median DT values were similar. The HFpEF group also achieved fewer METs and a lower peak heart rate and had a smaller HRR1. Thus, the lower HRRI cannot be attributed specifically to post-exercise deceleration.

### 3.3. Independent Association of PWV and HRRI with HFpEF

In univariable logistic regression analyses, higher PWV and lower HRRI were significantly associated with HFpEF (Table 4). The multivariable model included age, male sex, BMI, hypertension, type 2 diabetes mellitus, dyslipidemia, atrial fibrillation, PWV, and HRRI. Chronic kidney disease was excluded from the primary multivariable model because of complete separation. The analysis included all 180 participants, comprising 86 patients with HFpEF and 94 participants without HF.

In the multivariable analysis, both PWV and HRRI remained independently associated with HFpEF after adjustment for clinical characteristics. Each 1 m/s increase in PWV was associated with a 33.0% increase in the odds of HFpEF (adjusted OR 1.330, 95% CI 1.040–1.701; *p* = 0.023). Conversely, each 0.1-unit increase in HRRI was associated with a 14.3% reduction in the odds of HFpEF (adjusted OR 0.857, 95% CI 0.774–0.949; *p* = 0.003). Atrial fibrillation was also independently associated with HFpEF (adjusted OR 6.244, 95% CI 1.713–22.755; *p* = 0.006).

After incorporating beta-blocker use into the multivariable model, both PWV and HRRI remained independently associated with HFpEF, with estimates virtually unchanged from the primary model (PWV: adjusted OR 1.328, 95% CI 1.038–1.700, *p* = 0.024; HRRI: adjusted OR 0.857, 95% CI 0.774–0.949, *p* = 0.003). Beta-blocker use itself was not independently associated with HFpEF (adjusted OR 1.093, 95% CI 0.462–2.586, *p* = 0.840). The regression coefficients for PWV and HRRI changed by only −0.41% and 0.29%, respectively. These findings indicate that adjustment for recorded beta-blocker use did not materially alter the estimated associations of either marker with HFpEF (Table 5).

PWV and HRRI showed a moderate inverse correlation (Spearman’s ρ = −0.482, *p* < 0.001). Nevertheless, there was no evidence of problematic multicollinearity, with a maximum variance inflation factor of 1.972. The continuous predictors showed no evidence of departure from linearity in the logit according to the Box–Tidwell assessment. Model calibration was satisfactory, with no evidence of poor fit according to the Hosmer–Lemeshow test (*p* = 0.610). The final clinical + PWV + HRRI model had an apparent AUC of 0.8962. Across 1000 bootstrap resamples, all refitted models passed the reported convergence checks. Mean estimated optimism was 0.0267, yielding an optimism-corrected AUC of 0.870. Threshold selection was not repeated, and threshold-specific sensitivity and specificity were not internally validated.

To further assess the incremental contribution of PWV and HRRI to the clinical model, likelihood-ratio tests were performed using the same 180 participants across all nested models. Addition of PWV to the clinical model significantly improved model fit (likelihood-ratio χ^2^ = 10.945, *df* = 1; *p* < 0.001), as did addition of HRRI (likelihood-ratio χ^2^ = 15.201, *df* = 1; *p* < 0.001). The model incorporating both PWV and HRRI showed a significant improvement in fit compared with the clinical model alone (likelihood-ratio χ^2^ = 20.712, *df* = 2; *p* < 0.001). Moreover, addition of HRRI to the clinical + PWV model significantly improved model fit (likelihood-ratio χ^2^ = 9.768, *df* = 1; *p* = 0.002), while addition of PWV to the clinical + HRRI model also improved model fit (likelihood-ratio χ^2^ = 5.511, *df* = 1; *p* = 0.019). These findings provide evidence that PWV and HRRI each contributed information to the clinical model beyond the other biomarker.

Given the substantial difference in age between participants with and without HFpEF and the potential relationship between age and arterial stiffness, a sensitivity analysis was performed in which age was modelled using a restricted cubic spline with three knots. The spline model did not provide a significantly better fit than the model assuming a linear age effect (likelihood-ratio χ^2^ = 0.956, *df* = 1; *p* = 0.328). Importantly, the associations of both biomarkers with HFpEF remained essentially unchanged. In the spline-age model, each 1 m/s increase in PWV was associated with higher odds of HFpEF (adjusted OR 1.334, 95% CI 1.040–1.710; *p* = 0.023), while each 0.1-unit increase in HRRI was associated with lower odds of HFpEF (adjusted OR 0.867, 95% CI 0.781–0.962; *p* = 0.007).

### 3.4. Diagnostic Performance of PWV and HRRI

The discriminatory performance of PWV and HRRI for HFpEF was assessed using ROC curve analysis (Figure 3A). PWV demonstrated an area under the ROC curve (AUC) of 0.770 (95% CI, 0.702–0.838). The Youden index-derived threshold of ≥9.60 m/s yielded a sensitivity of 74.4% and a specificity of 69.2%. HRRI demonstrated an AUC of 0.792 (95% CI, 0.726–0.858), with an exploratory threshold of ≤1.97, yielding a sensitivity of 75.6% and a specificity of 74.5% (Table 6).

After exclusion of the 10 participants with chronic kidney disease, all of whom belonged to the HFpEF group, 170 participants remained (76 with HFpEF and 94 without HF). The AUC was 0.760 (95% CI, 0.689–0.832) for PWV and 0.777 (95% CI, 0.706–0.847) for HRRI, slightly lower than the corresponding full-cohort estimates.

In a sensitivity analysis restricted to the 153 participants with measured NT-proBNP, PWV yielded an AUC of 0.785 (95% bootstrap CI, 0.710–0.853) and HRRI an AUC of 0.795 (95% bootstrap CI, 0.723–0.863), similar to the full-cohort estimates.

The combined PWV + HRRI logistic regression model achieved an AUC of 0.833 (95% CI, 0.774–0.892). At the Youden index-derived predicted probability threshold of ≥0.343, the combined model yielded a sensitivity of 88.4% and a specificity of 64.9%. In paired DeLong comparisons with Holm adjustment, the combined PWV + HRRI model demonstrated significantly greater discrimination than PWV alone (adjusted *p* = 0.013) and HRRI alone (adjusted *p* = 0.021) (Figure 3A).

We next evaluated whether the addition of PWV and HRRI improved discrimination beyond a clinical model (Figure 3B). The clinical model, comprising age, male sex, BMI, hypertension, type 2 diabetes mellitus, dyslipidemia, and atrial fibrillation, yielded an AUC of 0.865 (95% CI, 0.811–0.919). Addition of PWV increased the AUC to 0.880 (95% CI, 0.830–0.930), whereas addition of HRRI increased the AUC to 0.885 (95% CI, 0.836–0.934). The model incorporating both PWV and HRRI had an AUC of 0.896 (95% CI, 0.851–0.942).

However, none of the biomarker-augmented clinical models demonstrated a statistically significant improvement in discrimination after Holm adjustment for the prespecified incremental clinical-model comparisons. Compared with the clinical model alone, addition of PWV yielded an adjusted *p* = 0.346, addition of HRRI yielded an adjusted *p* = 0.346, and addition of both PWV and HRRI yielded an adjusted *p* = 0.132. The model incorporating both biomarkers was also not significantly superior to either the clinical + PWV or clinical + HRRI model (adjusted *p* = 0.279 for both comparisons).

## 4. Discussion

PWV characterizes arterial stiffness, whereas HRRI integrates the timing of heart rate acceleration during exercise and deceleration during recovery. Their associations with HFpEF do not establish the mechanisms underlying these relationships [15].

Arterial stiffness has emerged as an important pathophysiological feature of HFpEF. Patients with HFpEF have been shown to exhibit PWV compared with individuals without heart failure, suggesting a greater degree of vascular stiffening and supporting the potential role of PWV as a marker of vascular dysfunction in this population [24,25,26].

Ultrasound-derived global PWV (gPWV, also termed PWVg) estimates arterial stiffness using Doppler-derived transit times between the aortic valve region and the common femoral artery. Studies in postmenopausal women reported increased PWVg and associations with left ventricular diastolic dysfunction and hypertrophy [27,28]. These observations support the relationship between arterial stiffening and ventricular dysfunction, but do not establish diagnostic accuracy for HFpEF or interchangeability with Arteriograph-derived PWV.

Routine blood-pressure and echocardiographic measurements can provide complementary information on arterial load. Effective arterial elastance is defined as end-systolic pressure divided by stroke volume (Ea = ESP/SV); using stroke volume indexed to body surface area yields the arterial elastance index (EaI = ESP/SVi). With ESP approximated as 0.9× brachial systolic blood pressure, this approach offers an accessible estimate of arterial load relevant to ventricular–arterial interaction [26,29]. Previous methodological work and an echocardiographic study in centenarians without overt cardiovascular disease illustrate the non-invasive assessment of arterial elastance and ventricular–arterial interaction [30,31]. However, Ea predominantly reflects vascular resistance and heart rate rather than arterial wall stiffness and should therefore be considered complementary to PWV [32]. Future studies could compare PWV with ESP/SVi-derived arterial elastance and test whether their combination improves HFpEF phenotyping or diagnostic discrimination beyond conventional clinical and echocardiographic variables.

Cozlac et al. [23] first introduced HRRI as a useful marker for assessing treatment response in patients undergoing cardiac resynchronization therapy. Subsequently, Dache et al. [33] demonstrated that this parameter may also serve as a valuable tool for the evaluation of patients with heart failure, particularly those with heart failure with preserved ejection fraction (HFpEF).

This cross-sectional study of 180 participants demonstrated that both PWV and HRRI were independently associated with clinically assigned HFpEF status after adjustment for age, sex, BMI, and major cardiovascular comorbidities, with exploratory, cohort-derived cutoffs of ≥9.60 m/s for PWV and ≤1.97 for HRRI. Both associations remained robust in multiple sensitivity analyses, including adjustment for beta-blocker use and modelling of age using restricted cubic splines, and the combined marker model significantly outperformed either marker in isolation.

The PWV threshold identified in this cohort was derived using the oscillometric Arteriograph device and should not be interpreted as equivalent to established carotid–femoral PWV thresholds obtained using a different measurement approach. The association between higher PWV and HFpEF is consistent with the growing conceptualization of HFpEF as a systemic disease extending beyond the myocardium to involve the vasculature [16], and it aligns with population-based longitudinal evidence linking estimated PWV to incident heart failure [18], as well as with a recent systematic review and meta-analysis confirming the association between arterial stiffness and heart failure across diverse populations [19]. The prognostic relevance of arterial stiffness for adverse cardiovascular outcomes independent of traditional risk factors, as demonstrated in the Framingham Heart Study [20], further supports the biological plausibility of our findings. Notably, arterial stiffness has been proposed to influence HFpEF pathophysiology through several interconnected pathways rather than a single mechanism, including increased pulse pressure, premature wave reflection, and impaired ventriculo-arterial coupling [21], which may partly explain the moderate, rather than excellent, discriminative performance of PWV in isolation (AUC 0.770).

Previous studies have described abnormal chronotropic responses and heart rate recovery in HFpEF [22,34,35]. However, HRRI incorporates both exercise and recovery timing. In Table S1, median acceleration time was shorter in the HFpEF group (334.5 versus 422 s), whereas median deceleration times were similar (185 versus 182 s). These descriptive findings highlight the exercise-phase contribution to the lower HRRI but neither establish equivalence of recovery times nor quantify the relative contributions of its components. HRRI should therefore not be interpreted as an isolated measure of autonomic recovery, and the mechanism underlying its association with HFpEF remains undetermined.

The moderate inverse correlation between PWV and HRRI (Spearman’s ρ = −0.482), together with their associations after mutual adjustment, supports their statistical complementarity but does not identify distinct underlying mechanisms. Although vascular-autonomic interactions have been described in hypertension [24], the present findings do not establish that autonomic dysfunction explains the association between HRRI and HFpEF.

The combined PWV + HRRI model showed greater apparent discrimination of clinically assigned HFpEF status than either marker alone, while likelihood-ratio testing indicated improved model fit. This pattern is consistent with the broader trend in HFpEF diagnostics toward multi-marker and multiparametric strategies, reflecting the pathophysiological heterogeneity of the syndrome and the recognition that no single marker, including natriuretic peptides, is sufficient to capture its full diagnostic and prognostic complexity [36].

The continued evolution of screening and diagnostic tools in this field, exemplified by the recent development of the HFpEF-ABA score, which uses only age, BMI, and history of atrial fibrillation to estimate HFpEF probability with a good discrimination (AUC 0.839), illustrates the ongoing effort to identify accessible, low-burden markers capable of improving diagnostic pathways [37]. Although adding PWV and HRRI improved model fit, the increase in AUC from 0.865 to 0.896 was not statistically significant after Holm adjustment (adjusted *p* = 0.132. Whether these markers provide clinical benefit, particularly in patients with intermediate HFA-PEFF or H2FPEF probability, remains a hypothesis requiring prospective evaluation; this subgroup was not directly assessed.

Given the well-documented chronotropic effects of beta-blockers, we performed a prespecified sensitivity analysis to evaluate their potential confounding influence on the HRRI-HFpEF association. Beta-blocker users exhibited numerically lower HRRI than non-users, although this difference did not reach statistical significance, and adjustment for beta-blocker use produced only negligible changes in the PWV and HRRI regression coefficients. The persistence of this association after adjustment for beta-blocker use does not establish an autonomic mechanism.

In a randomized, placebo-controlled trial in healthy young adults, acute beta1-blockade with bisoprolol paradoxically enhanced absolute heart rate recovery at 1 min compared with placebo, despite concurrently attenuating the chronotropic response during exercise [38]. This apparent paradox likely reflects the fact that beta-blockade lowers peak exercise heart rate, thereby reducing the absolute magnitude of the required decline during recovery, while potentially preserving or even facilitating the proportional return toward resting values.

From a clinical standpoint, both PWV and HRRI are inexpensive, widely available, and readily obtainable within routine cardiology assessment, in contrast to invasive hemodynamic evaluation or advanced strain-based echocardiographic techniques that remain less accessible in many centers. Their potential role within existing diagnostic pathways requires prospective evaluation, including in patients with intermediate diagnostic probability. Nevertheless, the thresholds identified here are exploratory, cohort-derived estimates, and their use to classify individual patients, especially those close to either threshold, cannot be recommended until measurement reliability and external diagnostic performance have been established [6,39].

### Limitations and Future Directions

Several limitations should be acknowledged. First, the cross-sectional, single-center design precludes causal inference and may limit the generalizability of the findings. The non-HF group was recruited from individuals undergoing cardiovascular evaluation rather than from a population-based healthy cohort, introducing the possibility of referral and selection bias. This comparator group may increase relevance to cardiovascular practice, however, the exploratory cutoffs reflect the selected clinical spectrum and require validation in patients presenting with suspected HFpEF.

Participants with HFpEF were substantially older than those without HF. Although age was included in the multivariable models and sensitivity analysis using restricted cubic splines yielded essentially unchanged associations for PWV and HRRI, residual confounding by age cannot be completely excluded. This concern is particularly relevant to PWV, and diagnostic performance among patients of comparable age remains to be established.

NT-proBNP was unavailable in 27/180 participants (15.0%), more often in the non-HF group, limiting complete HFA-PEFF assessment. A substantial proportion of participants with measured NT-proBNP had intermediate HFA-PEFF probability, and confirmatory diastolic stress echocardiography or invasive hemodynamic testing was not performed systematically. Group allocation was made by the treating cardiologist with access to PWV and HRRI, creating a potential for diagnostic review bias. Neither covariate adjustment nor restriction to participants with measured NT-proBNP removes the potential diagnostic review bias arising from the clinician’s access to PWV and HRRI results or provides an independent reference classification. Consequently, diagnostic misclassification cannot be excluded, and the discriminatory estimates and cohort-derived thresholds should be regarded as exploratory pending assessment against an independent, blinded reference classification.

Invasive hemodynamic assessment or diastolic stress echocardiography was not systematically performed in all participants; therefore, some degree of diagnostic misclassification, particularly among individuals with intermediate diagnostic probability, cannot be excluded.

The relatively modest sample size limited statistical power to detect small incremental improvements in discrimination when PWV and HRRI were added to an already well-performing clinical model. Chronic kidney disease was confined to the HFpEF group and could not be included in the primary multivariable model because of complete separation. Excluding these participants slightly reduced the marker AUCs, but residual confounding cannot be excluded.

Medication data were limited with respect to beta-blocker agent, dose, and treatment duration, precluding more detailed pharmacological sensitivity analyses. In addition, although adjustment for beta-blocker use did not materially alter the association between HRRI and HFpEF, residual effects of other medications influencing chronotropic response cannot be completely excluded.

PWV was assessed using an oscillometric Arteriograph device rather than conventional carotid–femoral PWV measurement; therefore, direct comparison of the derived PWV threshold with established carotid–femoral PWV reference values should be made cautiously. Furthermore, the PWV and HRRI cutoff values were derived and evaluated within the same cohort and should be considered exploratory until externally validated. Neither the thresholds nor their sensitivity and specificity were internally validated. Blood pressure measured specifically at PWV assessment was not retained in the analytical dataset. Its contribution to the observed between-group PWV differences could therefore not be assessed, and residual confounding by contemporaneous blood pressure cannot be excluded.

Measurement reliability is an additional limitation. Although at least two PWV recordings were obtained within a session, their consistency was judged by the operator without a predefined numerical tolerance, and quantitative within-session repeatability (intraclass correlation and absolute agreement) has not yet been established. HRRI acceleration and deceleration times were identified manually, but intraobserver and interobserver reading reproducibility has not yet been quantified. Importantly, no repeated exercise tests were performed, thus, the test–retest repeatability of HRRI cannot be assessed from this dataset.

The endpoint used to measure DT was determined by operator judgment without a prespecified numerical tolerance. Baseline heart rate, total exercise duration, peak workload in watts, and individual reasons for test termination were not consistently retained in the analytical dataset; only some original exercise reports remain available. Their group distributions therefore cannot be reported reliably. These limitations constrain the physiological interpretation and reproducibility of HRRI and reinforce the exploratory status of its cohort-derived threshold. Rate-adaptive pacemakers were substantially more frequent in participants with HFpEF than in non-HF comparators (23/86, 26.7%, versus 3/94, 3.2%). Device-mediated heart-rate responses during exercise and recovery may influence both components of HRRI. Individual programming parameters were unavailable for analysis, preventing assessment of the magnitude or direction of this potential influence. The imbalance in pacemaker prevalence therefore represents an additional potential source of confounding in the HRRI-HFpEF association.

Finally, HRRI was derived from a cycle ergometer protocol, and its reproducibility and applicability across different exercise modalities and testing protocols remain to be established. The potential utility of PWV and HRRI specifically among patients with intermediate HFA-PEFF or H2FPEF probability was not directly evaluated and warrants prospective investigation.

Future studies should prospectively validate the proposed PWV and HRRI thresholds in larger, multicenter cohorts and across different exercise-testing and arterial stiffness assessment protocols. Particular emphasis should be placed on patients with intermediate HFA-PEFF or H2FPEF probability, in whom their potential benefit remains to be established. Longitudinal studies are also needed to determine whether PWV and HRRI provide prognostic information beyond their cross-sectional association with HFpEF and whether serial changes in these parameters track disease progression or therapeutic response.

## 5. Conclusions

In this cross-sectional study, both PWV and HRRI were independently associated with clinically assigned HFpEF status after adjustment for age and major cardiovascular risk factors and comorbidities. Both markers showed moderate apparent standalone discrimination of the clinically assigned groups with AUCs of 0.770 for PWV and 0.792 for HRRI, while their combination significantly improved discrimination compared with either marker alone. Adding both markers improved clinical model fit, but incremental discrimination was not demonstrated after Holm adjustment (AUC 0.865 to 0.896; adjusted *p* = 0.132). The derived cutoff values of ≥9.60 m/s for PWV and ≤1.97 for HRRI are exploratory sample-specific estimates, not validated clinical decision thresholds. Quantitative measurement reliability and diagnostic performance against an independent, blinded reference standard remain to be established; these thresholds are unsuitable for individual clinical decision-making.

The associations remained robust in sensitivity analyses addressing beta-blocker use and alternative modelling of age. Combined assessment of PWV and HRRI provided greater discrimination than either marker alone. HRRI reflects both exercise and recovery timing, and its association with HFpEF should not be interpreted as evidence of impaired autonomic recovery.

Further prospective, multicenter studies are needed to validate these findings, assess their performance in patients with intermediate HFA-PEFF or H2FPEF probability, and determine whether PWV and HRRI also provide prognostic or treatment-response information over time.

## Figures and Tables

**Figure 1:**
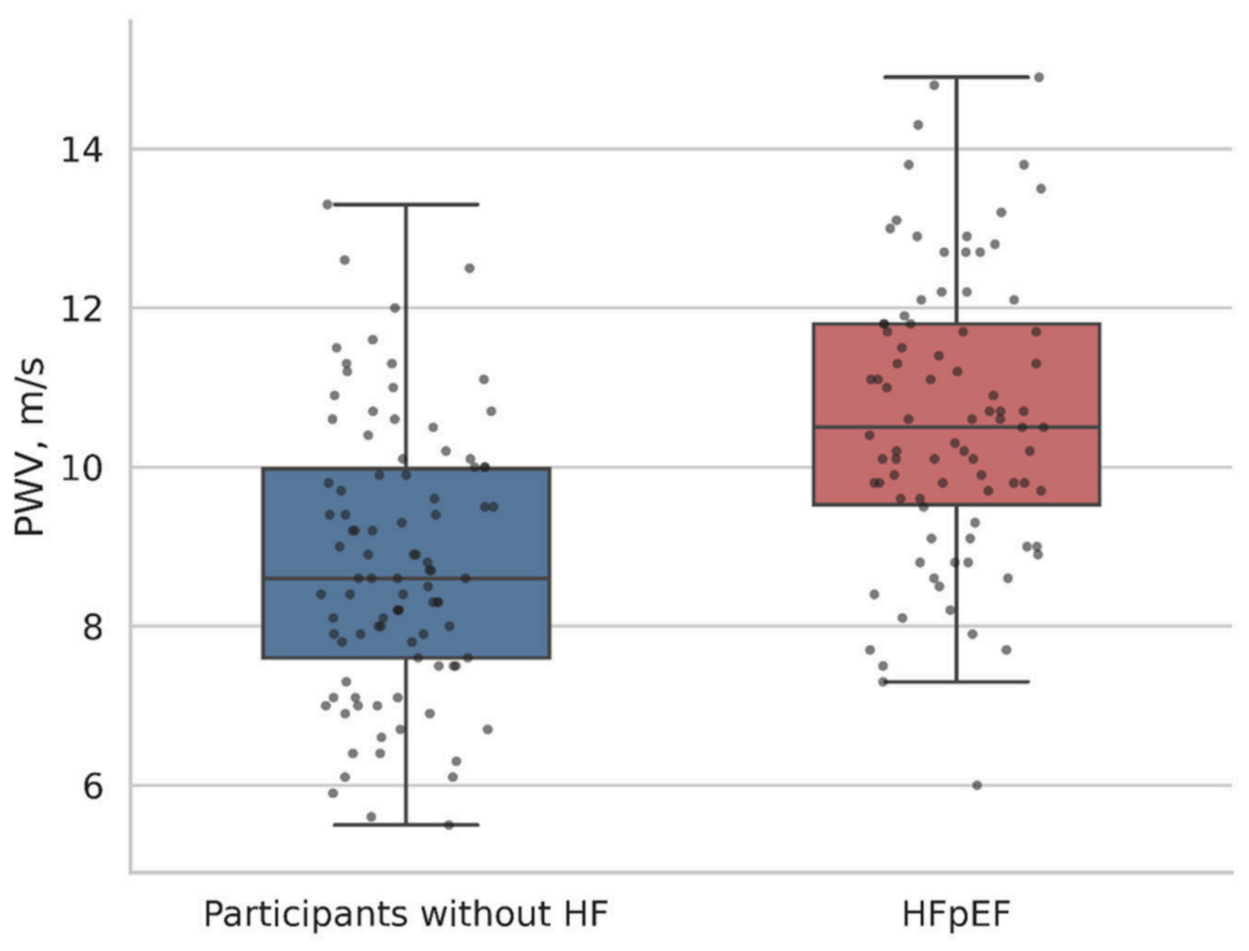
Pulse wave velocity according to HFpEF status. Boxplots show the median and interquartile range, with individual participant values overlaid. PWV was significantly higher in patients with HFpEF than in participants without HF (Mann–Whitney U test, *p* < 0.001).

**Figure 2:**
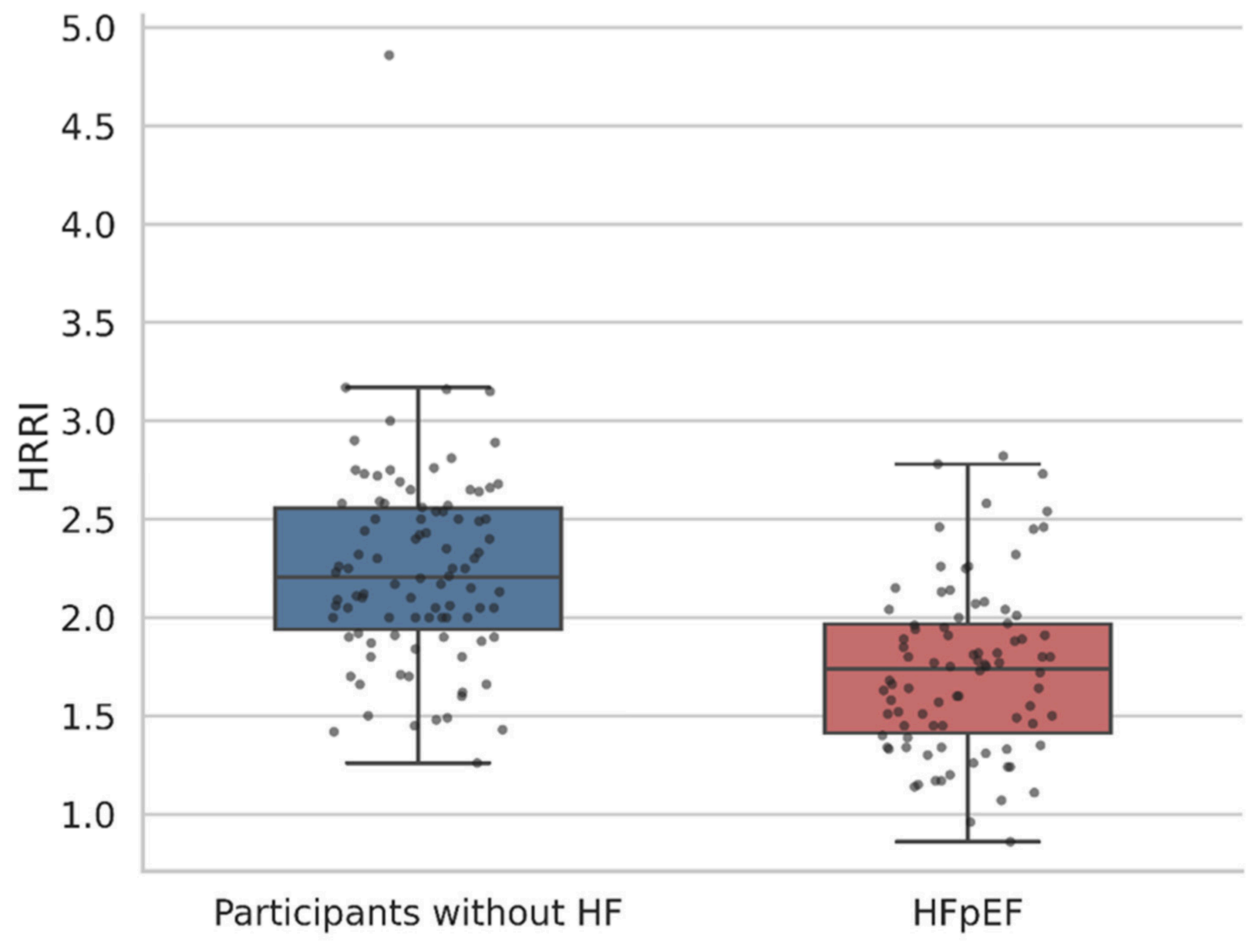
HRRI according to HFpEF status. Boxplots show the median and IQR, with individual participant values overlaid. HRRI was significantly lower in patients with HFpEF than in participants without HF (1.74 [1.41–1.97] vs. 2.21 [1.94–2.56], respectively; Mann–Whitney U test, *p* < 0.001).

**Figure 3:**
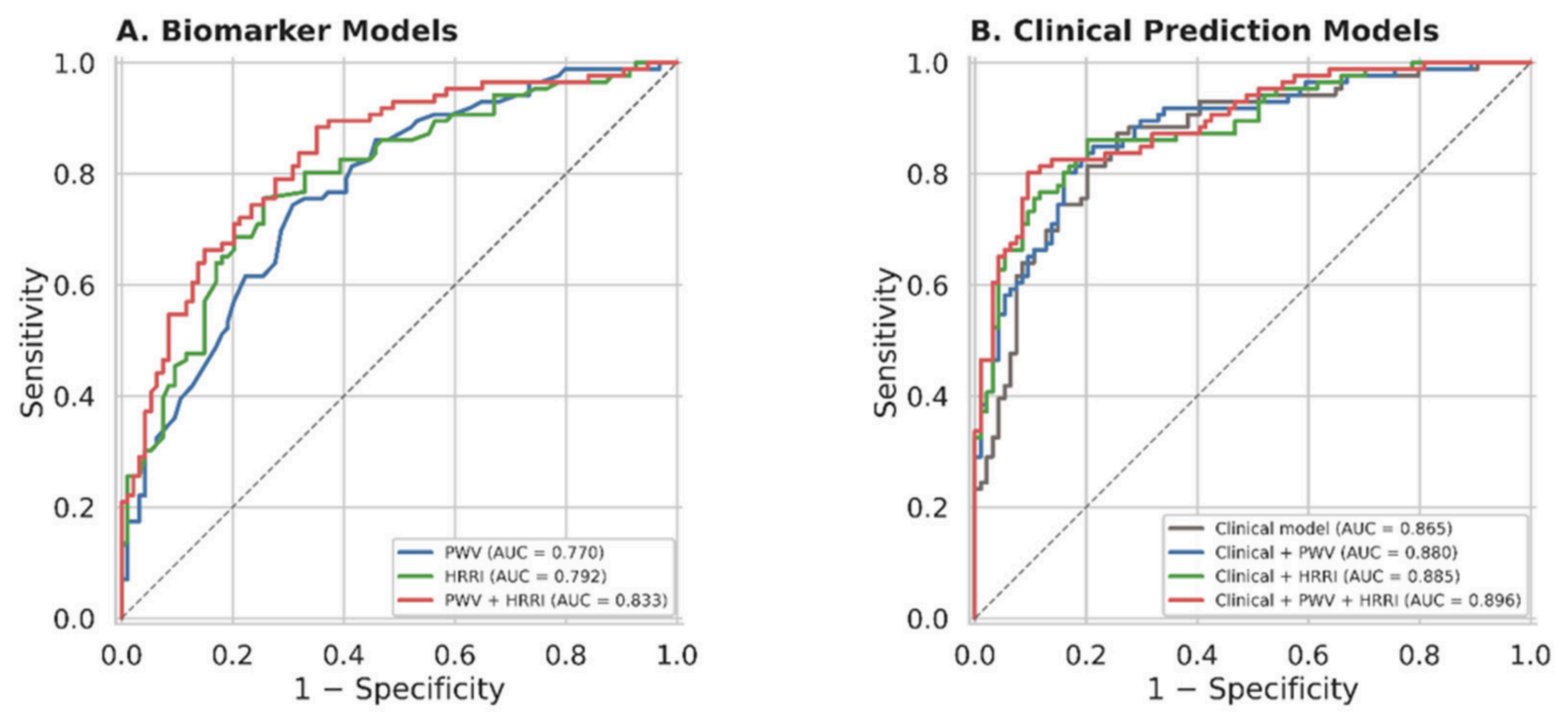
Receiver operating characteristic curves for discrimination of HFpEF. (**A**) Discriminatory performance of PWV, HRRI, and the combined PWV + HRRI model. (**B**) Discriminatory performance of the clinical model and clinical models incorporating PWV, HRRI, or both markers. The clinical model included age, male sex, BMI, hypertension, type 2 diabetes mellitus, dyslipidemia, and atrial fibrillation. The dashed diagonal lines represent the reference line of no discrimination (AUC = 0.5).

**Table 1:** Baseline characteristics of the study population.

Variable	Non-HF (*n* = 94)	HFpEF *(n* = 86)	*p*-Value
Age, years	48.50 [40.25–57.75]	64.00 [56.25–70.75]	<0.001
Male sex, (%)	63/94 (67.02%)	46/86 (53.49%)	0.064
BMI, kg/m^2^	27.80 [25.30–31.00]	28.90 [26.12–32.18]	0.124
Hypertension, (%)	57/94 (60.64%)	78/86 (90.70%)	<0.001
Type 2 diabetes mellitus, (%)	2/94 (2.13%)	19/86 (22.09%)	<0.001
Dyslipidemia, (%)	58/94 (61.70%)	77/86 (89.53%)	<0.001
Obesity, (%)	25/94 (26.60%)	35/85 (41.18%)	0.039
Atrial fibrillation, (%)	6/94 (6.38%)	23/86 (26.74%)	<0.001
Chronic kidney disease, (%)	0/94 (0.00%)	10/86 (11.63%)	<0.001
Beta-blocker users, (%)	46/94 (48.94%)	55/86 (63.95%)	0.043

**Table 2:** Objective diagnostic findings and HFA-PEFF categories by clinical group.

Variable	Non-HF (*n* = 94)	HFpEF (*n* = 86)
LVEF, %, median [IQR] (*n*)	55 [55–55] (94)	52 [50–55] (86)
LAVI, mL/m^2^, median [IQR] (*n*)	26.4 [20.0–32.4] (94)	34.1 [30.0–42.1] (86)
E/e′, median [IQR] (*n*)	7.0 [5.9–8.1] (94)	10.0 [8.3–12.0] (86)
NT-proBNP, pg/mL, median [IQR] (*n*)	50 [40–95] (71)	229 [135–459] (82)
NT-proBNP unavailable, *n* (%)	23 (24.5%)	4 (4.7%)
HFA-PEFF low probability, *n*/*N* (%)	39/71 (54.9%)	0/82 (0.0%)
HFA-PEFF intermediate probability, *n*/*N* (%)	32/71 (45.1%)	35/82 (42.7%)
HFA-PEFF high probability, *n*/*N* (%)	0/71 (0.0%)	47/82 (57.3%)
H_2_FPEF low probability (0–1), *n*/*N* (%)	34/94 (36.2%)	0/86 (0.0%)
H_2_FPEF intermediate probability (2–5), *n*/*N* (%)	60/94 (63.8%)	62/86 (72.1%)
H_2_FPEF high probability (6–9), *n*/*N* (%)	0/94 (0.0%)	24/86 (27.9%)

Values are median [IQR] with the number of available measurements in parentheses. HFA-PEFF categories are reported only for participants with measured NT-proBNP (71 non-HF and 82 HFpEF). The biomarker domain was unavailable for the remaining 27 participants, who were not assigned a complete HFA-PEFF category.

**Table 3:** Main study variables in participants with and without HFpEF.

Variable	Non-HF (*n* = 94)	HFpEF (*n* = 86)	*p*-Value
PWV, m/s	8.60 [7.60–9.98]	10.50 [9.53–11.80]	<0.001
HRRI	2.21 [1.94–2.56]	1.74 [1.41–1.97]	<0.001

**Table 4:** Univariable and multivariable logistic regression analyses for HFpEF.

Predictor	Univariable OR (95% CI)	*p* Value	Multivariable OR (95% CI)	*p*-Value
Age, per year	1.107 (1.071–1.143)	<0.001	1.059 (1.013–1.107)	0.011
Male sex	0.566 (0.309–1.035)	0.065	0.497 (0.203–1.216)	0.126
BMI, per kg/m^2^	1.061 (0.996–1.130)	0.067	1.073 (0.967–1.190)	0.186
Hypertension	6.329 (2.740–14.617)	<0.001	1.364 (0.417–4.460)	0.608
Type 2 diabetes mellitus	13.045 (2.938–57.918)	<0.001	2.591 (0.489–13.727)	0.263
Dyslipidemia	5.310 (2.372–11.890)	<0.001	1.996 (0.689–5.788)	0.203
Atrial fibrillation	5.354 (2.061–13.914)	<0.001	6.244 (1.713–22.755)	0.006
PWV, per 1 m/s	1.812 (1.473–2.229)	<0.001	1.330 (1.040–1.701)	0.023
HRRI, per 0.1-unit increase	0.773 (0.709–0.842)	<0.001	0.857 (0.774–0.949)	0.003

**Table 5:** Associations of PWV and HRRI with HFpEF before and after additional adjustment for beta-blocker use.

Predictor	Primary Model, Adjusted OR (95% CI)	*p* Value	Primary Model Plus Beta-Blocker Use, Adjusted OR (95% CI)	*p* Value
PWV, per 1 m/s increase	1.330 (1.040–1.701)	0.023	1.328 (1.038–1.700)	0.024
HRRI, per 0.1-unit increase	0.857 (0.774–0.949)	0.003	0.857 (0.774–0.949)	0.003
Beta-blocker use	Not included	—	1.093 (0.462–2.586)	0.840

The primary model included age, sex, body mass index, hypertension, type 2 diabetes mellitus, dyslipidemia, atrial fibrillation, PWV, and HRRI. The sensitivity model additionally included beta-blocker use. Only coefficients for PWV, HRRI, and beta-blocker use are displayed. Both models included 180 participants.

**Table 6:** Diagnostic performance of PWV, HRRI, and clinical prediction models for HFpEF.

Model/Marker	AUC (95% CI)	Exploratory Threshold	Sensitivity, % (95% CI)	Specificity, % (95% CI)
PWV	0.770 (0.702–0.838)	≥9.60 m/s	74.4 (64.3–82.5)	69.2 (59.2–77.6)
HRRI	0.792 (0.726–0.858)	≤1.97	75.6 (65.5–83.4)	74.5 (64.8–82.2)
PWV + HRRI	0.833 (0.774–0.892)	≥0.343 *	88.4 (79.9–93.6)	64.9 (54.8–73.8)
Clinical model	0.865 (0.811–0.919)	-	-	-
Clinical + PWV	0.880 (0.830–0.930)	-	-	-
Clinical + HRRI	0.885 (0.836–0.934)	-	-	-
Clinical + PWV + HRRI	0.896 (0.851–0.942)	-	-	-

* The threshold of 0.343 refers to the model-predicted probability from the combined PWV + HRRI logistic regression model. AUC CIs were calculated using the DeLong method. Sensitivity and specificity are presented with two-sided 95% Wilson score CIs without continuity correction. Exploratory thresholds for PWV, HRRI, and the PWV + HRRI model were selected using the Youden index in the same cohort in which performance was evaluated. These threshold-based estimates represent apparent performance and remain subject to optimism; their CIs do not account for threshold-selection or model-development uncertainty.

## Data Availability

The original contributions presented in this study are included in this article. Further inquiries can be directed to the corresponding author.

## Supplementary Materials

The following supporting information can be downloaded at: https://www.mdpi.com/article/10.3390/jcm15218363/s1, Table S1: Exercise-test characteristics by study group.

## Abbreviations

The following abbreviations are used in this manuscript:

A	Late diastolic trans-mitral flow velocity
APMHR	Age-predicted maximal heart rate
AT	Acceleration time
AUC	Area under the receiver operating characteristic curve
BMI	Body mass index
cfPWV	Carotid–femoral pulse wave velocity
CI	Confidence interval
df	Degrees of freedom
DT	Deceleration time
E	Early diastolic trans-mitral flow velocity
e′	Early diastolic mitral annular velocity
E/A	Ratio of early to late diastolic trans-mitral flow velocities
E/e′	Ratio of early diastolic trans-mitral flow velocity to early diastolic mitral annular velocity
ESC	European Society of Cardiology
HF	Heart failure
HFA-PEFF	Heart Failure Association diagnostic algorithm incorporating Pre-test assessment, Echocardiography and natriuretic peptides, Functional testing, and Final etiology
H_2_FPEF	Heavy, Hypertensive, atrial Fibrillation, Pulmonary hypertension, Elder, and Filling pressure
HFpEF	Heart failure with preserved ejection fraction
HFpEF-ABA	HFpEF probability score based on Age, Body mass index, and Atrial fibrillation
HFrEF	Heart failure with reduced ejection fraction
HRR	Heart rate recovery
HRR1	Heart rate recovery at 1 min
HRRI	Heart rate recovery index
IQR	Interquartile range
LAVI	Left atrial volume index
LVEF	Left ventricular ejection fraction
MAPSE	Mitral annular plane systolic excursion
METs	Metabolic equivalents
NT-proBNP	N-terminal pro-B-type natriuretic peptide
NYHA	New York Heart Association
OR	Odds ratio
PWV	Pulse wave velocity
ROC	Receiver operating characteristic
VIF	Variance inflation factor
