# Supplementary material for: Heart Rate Recovery Index and Pulse Wave Velocity in HFpEF: Exploratory Cutoff Values from a Cross-Sectional Study
Source: J Clin Med. 2026 Oct 27;15(21):8363. doi: 10.3390/jcm15218363 (PMC13763607; doi:10.3390/jcm15218363)
Supplement: Supplementary file 1 [file jcm-15-08363-s001.zip › jcm-4613789-supplementary.pdf]

Supplementary Materials

Table S1. Exercise-test characteristics by study group

Variable, median [IQR]	Non-HF (*n* = 94)	HFpEF (*n* = 86)
AT, s	422 [344–500.3]	334.5 [281.3–399.8]
DT, s	182 [150–210]	185 [180–236.3]
HRRI	2.21 [1.94–2.56]	1.74 [1.41–1.97]
Achieved exercise capacity, METs	9.20 [8.00–10.55]	7.55 [6.50–8.38]
Peak heart rate, beats/min	132.5 [118–146]	111.5 [100–122.5]
HRR1, beats/min	25 [20–30.8] (n = 94)	16 [13–20] (n = 83)
Implanted rate-adaptive pacemaker, n (%)	3 (3.2%)	23 (26.7%)
